# Two new bee-killing flies from Brazil (Insecta: Diptera: Phoridae: *Melaloncha*)

**DOI:** 10.3897/BDJ.4.e7715

**Published:** 2016-02-08

**Authors:** Brian V. Brown

**Affiliations:** ‡ Natural History Museum of Los Angeles County, Los Angeles, United States of America

**Keywords:** Phoridae, taxonomy, Neotropical, parasitoid

## Abstract

**Background:**

The genus *Melaloncha* is a large group of species of parasitoid phorid flies that attack Hymenoptera, mostly stingless bees (Meliponinae, Apidae) in the Neotropical Region.

**New information:**

Two new Brazilian species, Melaloncha (Melaloncha) peacockorum sp. n. and Melaloncha (Udamochiras) nielsi sp. n., are described and their identification clarified.

## Introduction

Bee-killing flies of the genus *Melaloncha* are small (1.5-4.5 mm) fast, agile parasitoids, mostly of stingless bees, bumble bees, and honey bees, but with records also from orchid bees, sweat bees, and vespid wasps (Ament et al. 2013, Brown 2006, Lucia et al. 2013, Lutz and Brown 2013, Ramírez 1982, Wcislo et al. 2004, Brown 2001). Female flies attack hosts by injecting an egg into their body through a membranous area between exoskeleton plates, and the resulting larvae consume and kill them. These flies have been studied in detail recently, with over 150 new species described (Braet 2013, Brown 2004a, Brown 2005, Brown 2006, Brown and Kung 2006, Brown 2004b, Brown 2009, Gonzalez and Brown 2004, Kung 2008) and phylogenetic relationships hypothesized (Brown and Smith 2010, Smith and Brown 2008). A total of 170 species are now recognized (exclusive of the two described in this paper), but it is likely that many more remain to be found.

## Materials and methods

Specimens were borrowed from the collections of the Instituto Nacional de Pesquisas da Amazônia, Manaus, Brazil (INPA) and the Universidade de São Paulo, Ribeirão Preto, Brazil. Photographs were taken using a Keyence V5000 digital microscope.

## Taxon treatments

### Melaloncha (Melaloncha) peacockorum sp. n.

urn:lsid:zoobank.org:act:FE39F86E-1F6C-4A29-8F8E-A8A6B9F4A49C

#### Materials

- **Type status:** Holotype. **Occurrence:** catalogNumber: LACM ENT 335990; recordedBy: Amorim, Ribeiro, Berbert; sex: female; lifeStage: adult; **Location:** country: Brazil; stateProvince: SP; locality: Reserva Biológica Boracéia; verbatimLatitude: 23°39'S; verbatimLongitude: 45°53'W; verbatimCoordinateSystem: degrees minutes; **Event:** samplingProtocol: Shannon trap; eventDate: 2009-11-20/25; verbatimEventDate: 20-25 November 2009; **Record Level:** type: PhysicalObject; institutionCode: LACM; collectionCode: ENT; ownerInstitutionCode: MZSP; basisOfRecord: PreservedSpecimen

#### Description

**Figure 1.**
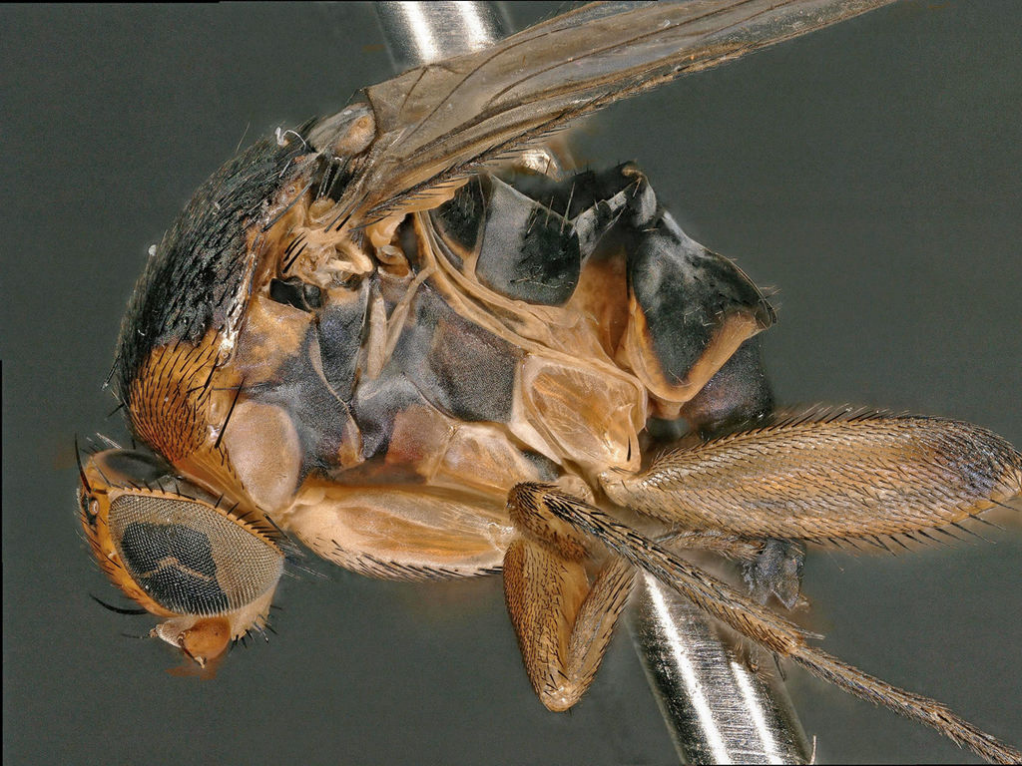
Habitus, left lateral, of Melaloncha (Melaloncha) peacockorum, new species

**Figure 2.**
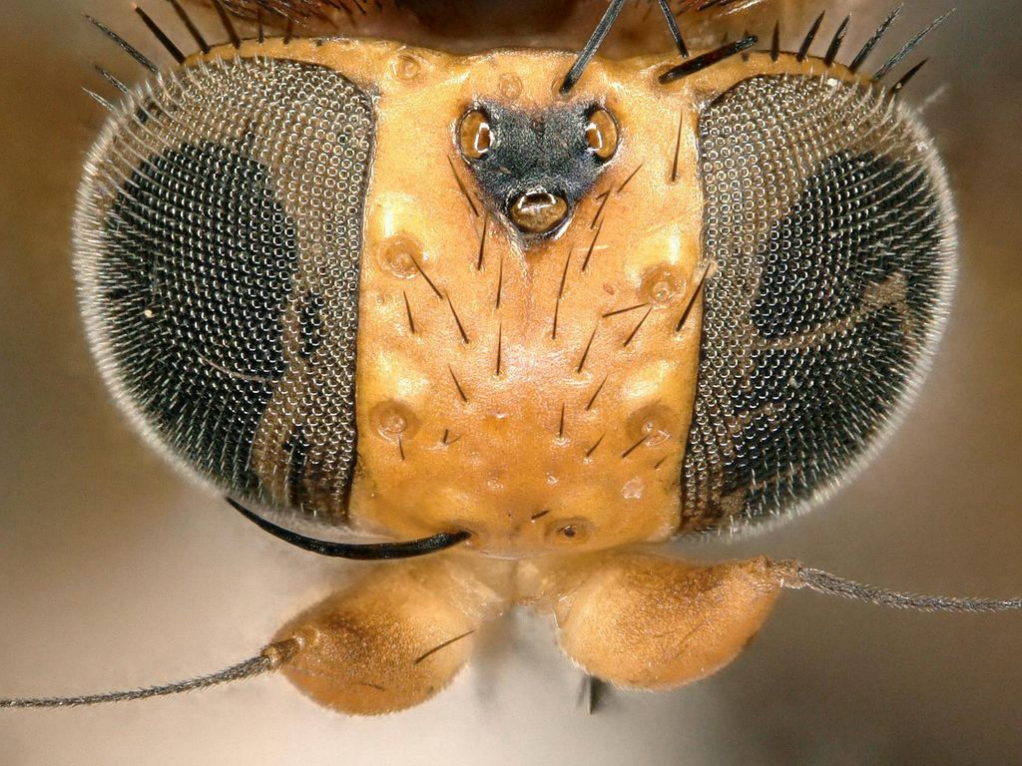
Head, anterior, of Melaloncha (Melaloncha) peacockorum new species

**Figure 3.**
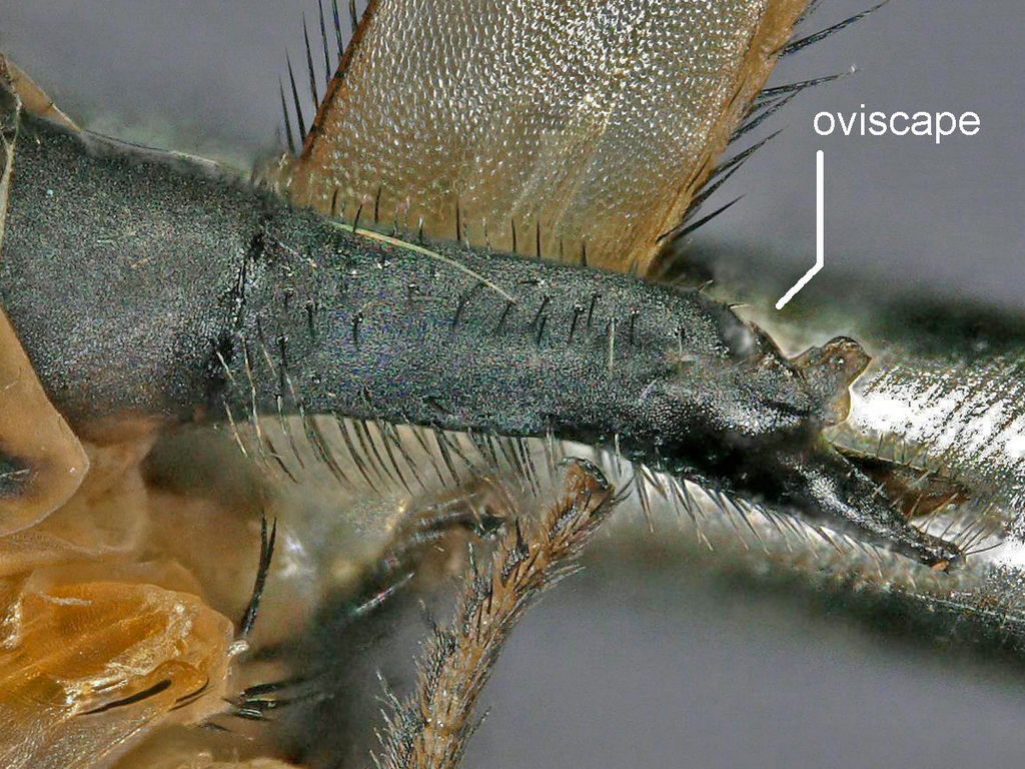
Oviscape, left lateral, of Melaloncha (Melaloncha) peacockorum new species

**Figure 4.**
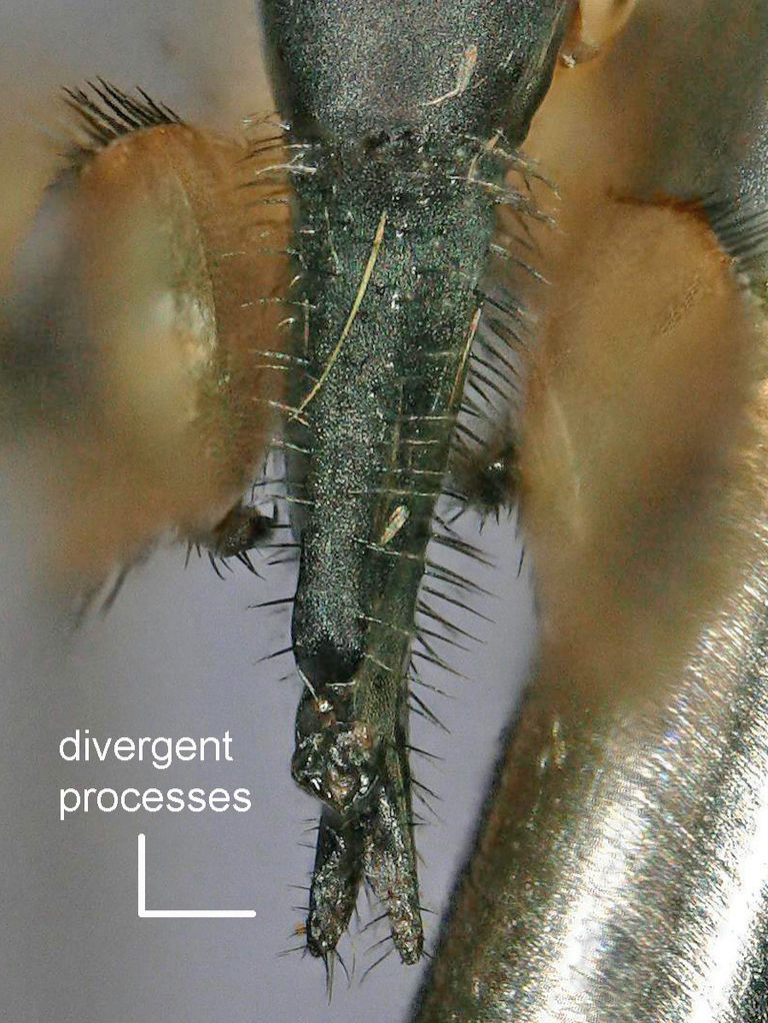
Oviscape, dorsal, of Melaloncha (Melaloncha) peacockorum new species

Female (Figs 1, 2, 3, 4). Body length approximately 3.0 mm. Frons orange, except ocellar triangle black; sculpturing finely reticulate with numerous punctures, most bearing setae. Frons 0.38 head width. Dorsal interfrontal setae absent. Flagellomere 1 orange. Palpus whitish-yellow, with long, black setae. Dorsal postocular setae black; genal and other postocular setae black. Scutum dark (Fig. 3), except anterolaterally yellow. Anterior scutellar seta long, thick, posterior scutellar setae missing from specimen. Pleuron various colors, from yellow to black. Legs yellow. Foretibia with irregular dorsal bare area. Foretarsomeres unmodified. Posterior claw of foreleg not enlarged, claws lobed at base. Costa 0.52 wing length. Wing vein R2+3 absent. Halter yellow. Abdominal tergites black with silvery iridescence. Venter of abdomen yellow. Oviscape black, setose, with rounded, shorter dorsal lobe and longer ventral lobe ending in pair of divergent processes.

#### Diagnosis

Dark colored Melaloncha (Melaloncha) with wide, orange, punctate frons, and oviscape with blunt, dorsal, median lobe plus more ventral, bifurcate lobe. In the most recent key to Melaloncha (Melaloncha) species (Brown 2006), *M. peacockorum* does not successfully pass couplets 5 to 6, which should be modified as follows:

5. Oviscape with apical pair of divergent, narrow setose processes... .5a

- Oviscape lacking setose processes, or, if present, such processes arising ventrally and anterior to apex (as in oviscapes of the *M. cingulata* group- Brown 2004b)... ..6

5a. Setae of divergent apical processes long, curved, dorsally directed (fig. 32 in Brown 2006); body color mostly yellow..... *M. ciliata* Brown

- Setae of apical processes shorter, straight, not predominantly dorsally directed; body color mostly dark... . *M. peacockorum* n. sp.

#### Etymology

Named for the Peacock family at the request of Jon Peacock, a supporter of the Entomology Department of the Natural History Museum of Los Angeles County (LACM).

#### Distribution

Brazil.

#### Ecology

Like most *Melaloncha* species, this fly is probably a parasitoid of stingless bees. It was collected with a Shannon trap, whose operation I have observed in Brazil. The trap is a large structure consisting of a square of black netting, about 3 m in length on each side, with outer walls of the same material. In construction it is like a large box missing the bottom side. The trap is suspended so that the sides are about 0.3 m above the ground, allowing insects access to the bait. Many insects attempt to escape by flying upwards, rather than using the small opening at ground level, and thus get caught in the top of the trap.

The bait used by the researchers is placed in a shallow pit near the center, and consists of a couple of fish, chicken meat, various vegetables, a bag of oatmeal, some mushrooms, human feces, and urine. This smorgasborg "ripens" over several days and attracts hordes of flies, but also other insects including stingless bees, which are frequently attracted to protein (Roubik 1989). Probably, an aggregation of such bees attracted this fly.

### Melaloncha (Udamochiras) nielsi sp. n.

urn:lsid:zoobank.org:act:D20C44D2-00C4-457F-945A-95461C290F15

#### Materials

- **Type status:** Holotype. **Occurrence:** catalogNumber: LACM ENT 335989; recordedBy: A. Henriques; sex: female; lifeStage: adult; **Location:** country: Brazil; stateProvince: AM; verbatimLocality: Manaus, Reserva Ducke, Igarapé Barro Branco; locationRemarks: 20m above forest floor; **Event:** samplingProtocol: Arm. Suspensa; eventDate: 2004-11-8/18; verbatimEventDate: 08-18.xi.2004; **Record Level:** institutionID: INPA; ownerInstitutionCode: INPA; basisOfRecord: PreservedSpecimen

#### Description

**Figure 5.**
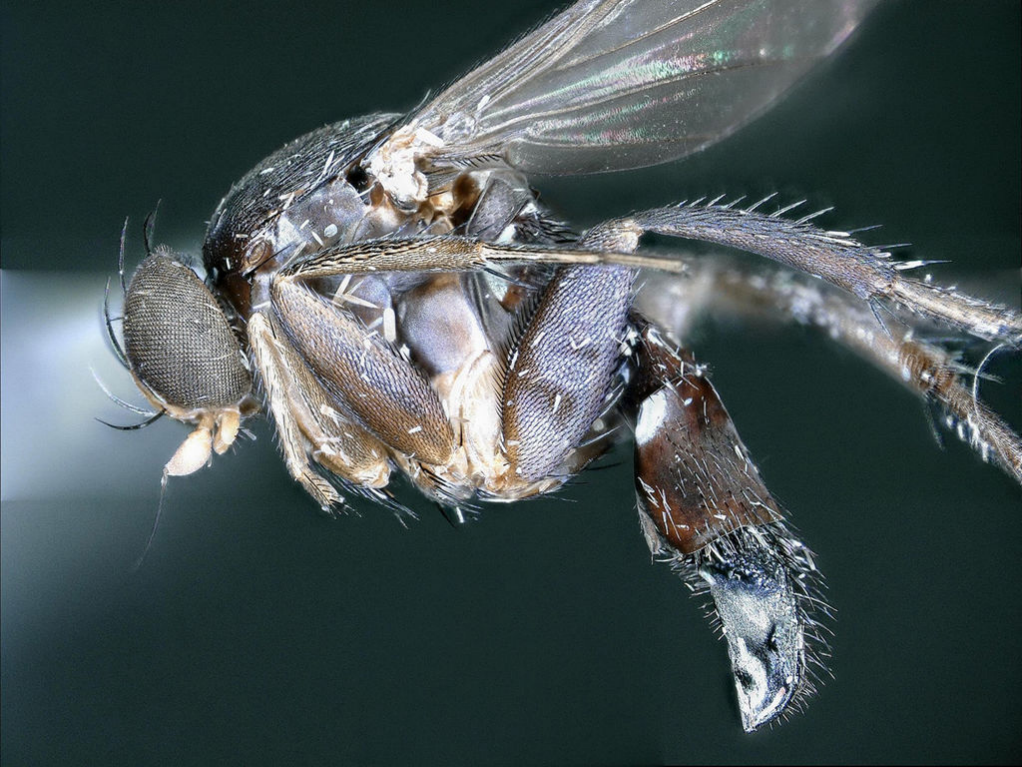
Habitus, left lateral, of Melaloncha (Udamochiras) nielsi sp. n.

**Figure 6.**
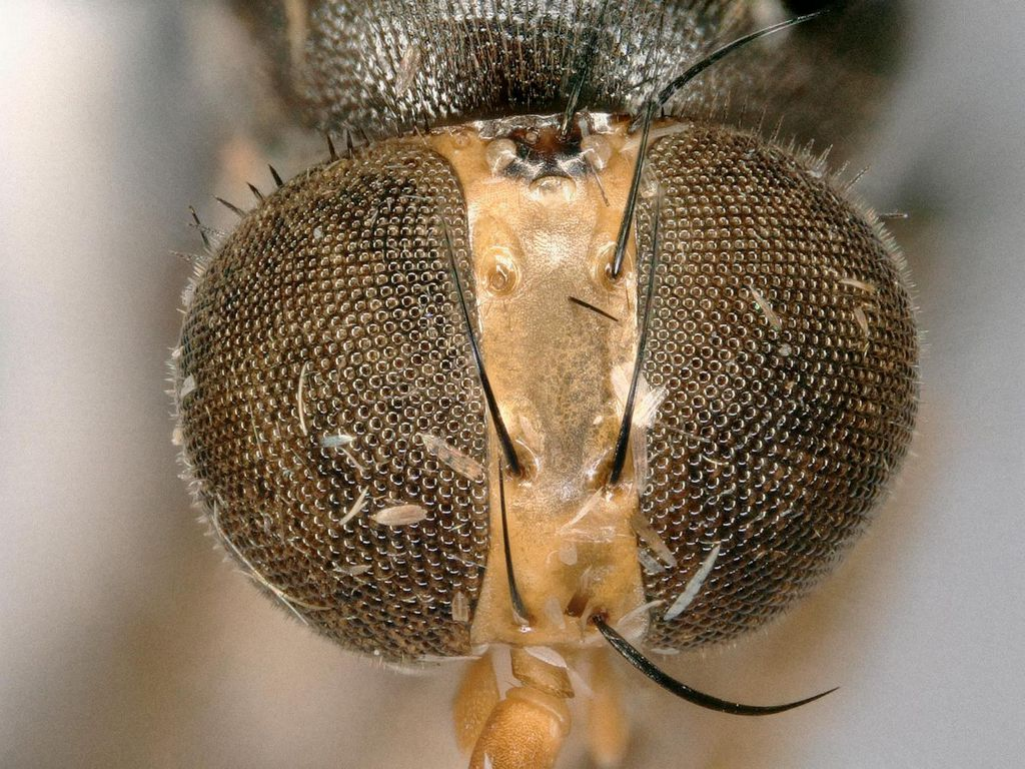
Head, anterior, of Melaloncha (Udamochiras) nielsi sp. n.

**Figure 7.**
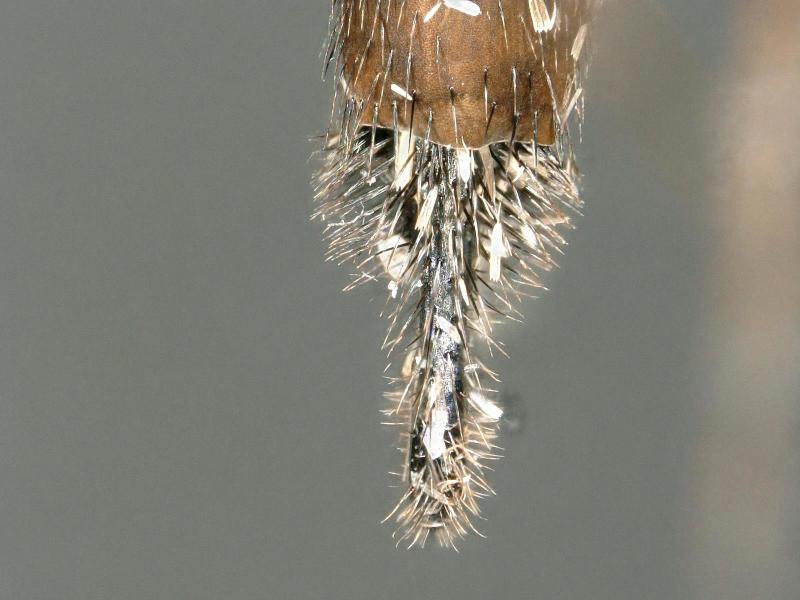
Oviscape, dorsal, of Melaloncha (Udamochiras) nielsi sp. n.

Female (Figs 5, 6, 7). Body length approximately 3.8 mm. Frons orange, except ocellar triangle black; sculpturing finely reticulate with numerous lateral punctures, most bearing setae. Frons 0.22 head width. Dorsal interfrontal setae absent. Flagellomere 1 yellow. Palpus yellow, with black setae. Dorsal postocular setae black; genal and other postocular setae black. Thorax mostly black. Anterior scutellar seta long, thin, posterior scutellar seta extremely long. Legs brown, mid- and hind legs dark brown. Foretibia with irregular dorsal bare area. Foretarsomeres missing from both legs. Claws visible only on one hind leg, apically bifurcate. Costa 0.61 wing length. Wing vein R2+3 absent. Halter brown. Abdominal tergites brown with silvery iridescence. Venter of abdomen gray. Oviscape black, with dense, strong black setae basally, dorsally, and ventrally; apically laterally flattened, dorsal surface curved ventrally, ventral surface straight.

#### Diagnosis

Large dark species with oviscape densely setose at base, laterally flattened, dorsal surface curved ventrally. In the key to species (Brown 2004a), *M. nielsi* comes closest to *M. valeria* Brown, from which it differs by the dense black setae (absent from *M. valeria*) and greatly compressed structure of the oviscape. It does not resemble any of the more recently described species of Melaloncha (Udamochiras) (Gonzalez and Brown 2004, Brown 2009, Braet 2013).

#### Etymology

Named for Niels Jensen at the request of Sara Jensen, a supporter of the Entomology Department of the Natural History Museum of Los Angeles County.

#### Distribution

Brazil

#### Ecology

Unknown, but presumably parasitoids of stingless bees like most other *Melaloncha* species.

## Supplementary Material

XML Treatment for Melaloncha (Melaloncha) peacockorum

XML Treatment for Melaloncha (Udamochiras) nielsi
